# What is the Etiology of Dysarthria and Ataxia in a Woman With Cancer?

**DOI:** 10.6004/jadpro.2014.5.2.2

**Published:** 2014-03-01

**Authors:** Paula J. Anastasia

**Affiliations:** From Cedars-Sinai Medical Center, Los Angeles, California

## History

R. J. is a 56-year-old woman with recurrent stage 1a, grade 3 endometrial carcinoma who was first diagnosed in 2011. She underwent hysterectomy and bilateral salpingo-oophorectomy with lymph node dissection. No adjuvant therapy was recommended, and she was followed every 3 months. Recurrent endometrial cancer was diagnosed in June 2012 after a left cervical lymph node biopsy showed metastatic adenocarcinoma consistent with endometrial primary. A CT of the abdomen and pelvis showed metastatic disease to the periaortic lymph nodes and omentum.

R. J. began IV carboplatin and paclitaxel every 3 weeks. She completed 5 cycles of chemotherapy from June to October 2012. During this time she ruptured her Achilles tendon. She required surgery followed by IV antibiotics for a postoperative infection. She developed a deep-vein thrombosis of the lower extremity, which is managed with warfarin.

## Chief Complaint

In November 2012, R. J.’s partner calls the advanced practitioner to report that R. J. is exhibiting gait imbalance and slurred speech. The team is told for the first time that R. J. had been experiencing mild, intermittent lightheadedness with increasing unsteadiness and gait imbalance since September, but now these symptoms are becoming pronounced.

Because brain metastasis is suspected, the team has R. J. come in to the facility for a brain CT; no evidence of infarction, hemorrhage, or metastatic disease is seen. A PET/CT that had been done in October showed several sites of stable disease in the peritoneal cavity, consistent with her recurrent endometrial cancer. After her Achilles surgery, R. J. had been walking with crutches but later used a walker; as of now, she is in a wheelchair most of the time. She occasionally has double vision, but no opsoclonus or nystagmus. She denies headache, nausea, seizures, or difficulty swallowing. She has no shortness of breath and no incontinence.

## Physical Examination and Diagnostic Studies

After the brain CT, R. J. comes into the office for an evaluation. Cranial nerves are within normal limits; strength is 5/5; gait is wide based, but R. J. needs assistance to ambulate (this is partially due to her past Achilles tendon surgery). She cannot perform the Romberg test and would fall without steadying assistance. Her tone and sensory exam is normal. Upon coordination review she demonstrates a slowing of rapid alternating movement but good finger-nose-finger and heel-shin coordination. Deep-tendon reflexes are as follows: 2+ biceps, absent triceps and brachial radialis, 2+ patellas, absent ankle jerks and silent plantar responses. R. J.’s cognitive function is intact. Comprehensive chemistry panel and complete blood count are normal. As part of her workup, a serum paraneoplastic panel is ordered, showing positive anti-Yo, negative anti-MAG, and negative anti-Hu Western Blot (see Table).

**Table 1:**
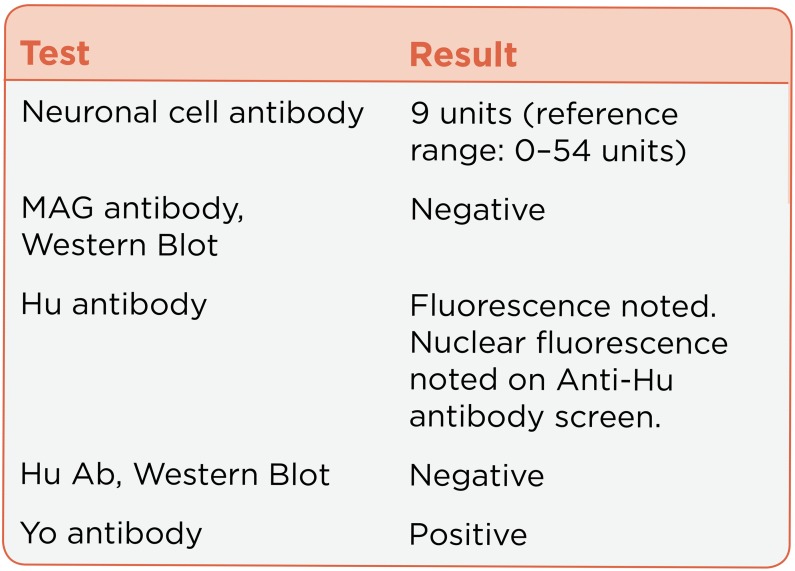

She is referred to a neurologist, who orders a brain MRI to rule out abnormality. It shows atrophy of the cerebellum and enlargement of the third and lateral ventricles but no evidence of metastatic disease or infarction. R. J.’s medication list includes oral warfarin 5 mg and bupropion 150 mg daily for depression.

**Figure 1:**
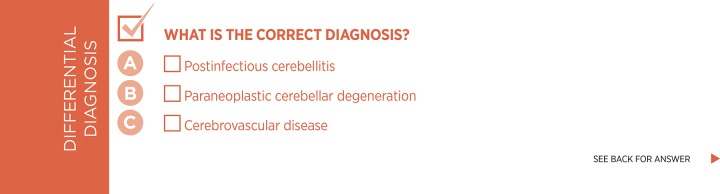
What is the correct diagnosis?

## Correct Answer: B

**Paraneoplastic cerebellar degeneration (PCD) **is a rare paraneoplastic syndrome associated with lung and gynecologic cancers as well as Hodgkin disease. Neurologic symptoms often occur prior to a cancer diagnosis (Russo et al., 2013). These include dizziness, nausea, gait instability, diplopia, ataxia, and dysarthria. Symptoms may present gradually and progress rapidly for about 6 months before becoming debilitating (Finsterer, Voigtländer, & Griswold, 2011). The clinical cerebellar ataxia evident in patients with PCD is caused by Purkinje neuronal loss in the cerebellum (Dalmau & Rosenfeld, 2008).

The etiology of PCD is considered to be an autoimmune reaction aimed against the Purkinje cells in the cerebellum. It is thought to be initiated when tumor cells such as those associated with ovarian or breast cancer express the Purkinje neuron protein (cdr2) normally expressed in the brain (Dalmau & Rosenfeld, 2008). Thus, an antitumor immune response occurs in addition to an antineuronal immune response. Patients with PCD will test positive for an antineuronal antibody known as anti-Yo, which destroys the Purkinje cells, causing the clinical picture of PCD. Since the criteria for diagnosing PCD had been met for R. J. (classic syndrome, positive antibody, and corresponding malignancy), cerebrospinal fluid did not need to be obtained.

## Explanation of Incorrect Answers

**Postinfectious cerebellitis** is characterized by an onset of ataxia after a viral infection such as chickenpox, mycoplasma pneumonia, or Epstein-Barr virus. It is often seen in children, but it has been seen in adults (Gruis et al., 2003). Symptoms include ataxia characterized by clumsy body movements, nausea, and headaches. R. J. had a bacterial infection after her Achilles surgery but no viral infection. Infectious cerebellitis does not manifest with dysarthria, and the ataxia usually resolves with no treatment (Dalmau & Rosenfeld, 2008).

**Cerebrovascular disease (CVD) **is a category of brain dysfunction related to the blood vessels that supply the brain. Risk factors for CVD include hypertension, diabetes, smoking, and ischemic heart disease. R. J. had no obvious risk factors for CVD, yet a stroke or transient ischemic attack can cause facial weakness, visual impairment, gait imbalance, and dysphasia (Savitz & Mattle, 2013). Yet patients with CVD generally have some degree of mental confusion, which R. J. did not exhibit. Cerebral thrombosis or embolism can cause a stroke, but this would result from a rupture of a blood vessel or a blockage in the brain. The MRI did not confirm the presence of either of these events.

## Treatment

Unfortunately, patients with PCD associated with anti-Yo antibodies have a poor prognosis and are less likely to recover from their illness. Traditionally, PCD treatment has consisted of high-dose steroids and plasmapheresis or IVIG (IV immunoglobulin). Anecdotal reports show promising efficacy with IV rituximab (Rituxan), an anti-CD20 antibody (Esposito et al., 2008). R. J. was treated with daily IVIG for 5 days followed by 8 weekly treatments with rituximab. Subsequently, paclitaxel and carboplatin IV every 3 weeks was resumed to manage her endometrial cancer. One report (Phuphanich & Brock, 2007) showed neurologic improvement with IVIG every 4 to 6 weeks followed by chemotherapy to treat the underlying malignancy, similar to what was prescribed for R. J.

## Follow-up

Immediately after R. J. received her combination modalities of IVIG, high-dose methylprednisone, rituximab, and paclitaxel plus carboplatin, there was an improvement in her dizziness and speech. Her balance was still poor, but she was able to stand comfortably with two-person assistance. Her cranial nerves remained intact, as did her cognitive function. R. J. received occupational and physical therapy. By the second month of treatment R. J. was able to ambulate with a walker and articulate words. Unfortunately, her progress was short lived. The neurologic degeneration began accelerating, despite treatment. R. J.’s overall weakness returned, and she was dependent on others for care. Although her endometrial cancer appeared stable, her performance status was deteriorating. The headaches returned, and her speech became more slurred. After a discussion involving the multidisciplinary team and R. J. and her family members, R. J. agreed to receive palliative and supportive care at home.

## References

[1] Dalmau Josep, Rosenfeld Myrna R (2008). Paraneoplastic syndromes of the CNS.. *Lancet neurology*.

[2] Esposito Marcello, Penza P, Orefice G, Pagano A, Parente E, Abbadessa A, Bonavita V (2008). Successful treatment of paraneoplastic cerebellar degeneration with Rituximab.. *Journal of neuro-oncology*.

[3] Finsterer Josef, Voigtländer Till, Grisold Wolfgang (2011). Deterioration of anti-Yo-associated paraneoplastic cerebellar degeneration.. *Journal of the neurological sciences*.

[4] Gruis Kirsten L, Moretti Paolo, Gebarski Stephen S, Mikol Daniel D (2003). Cerebellitis in an adult with abnormal magnetic resonance imaging findings prior to the onset of ataxia.. *Archives of neurology*.

[5] Phuphanich Surasak, Brock Charles (2007). Neurologic improvement after high-dose intravenous immunoglobulin therapy in patients with paraneoplastic cerebellar degeneration associated with anti-Purkinje cell antibody.. *Journal of neuro-oncology*.

[6] Russo Alessia Erika, Scalone Simona, Leonardi Giulia Costanza, Scalisi Aurora, Giorda Giorgio, Sorio Roberto (2013). Paraneoplastic cerebellar degeneration associated with ovarian cancer.. *Oncology letters*.

[7] Savitz Sean I, Mattle Heinrich P (2013). Advances in stroke: emerging therapies.. *Stroke; a journal of cerebral circulation*.

